# 

**Published:** 1889-09-07

**Authors:** 


No. 154, Vol. VI.—September 7th, 1889.
Registered at the General Post Office as a Newspaper.]

Monthly Parts, 6d.; post free 7½d.

Ditto per Annum, 7s. 6d., post free.

